# Obituary

**Published:** 1905-02-15

**Authors:** 


### OBITUARY.

DR. ELI T. STARR, formerly in the employ of the S. S. White Dental Manufacturing Company, died at the home of his daughter, Mrs. John Jolly, of Bala, Pa., December

11th, 1904. Dr. Starr was connected with the S. S. White Company for nearly forty years, and was one of the most valuable men connected with the development of the varied business relations of this house and the dental profession. His work was that of putting in merchandizable form the inventions of the profession. He was a skillful mechanic and a man of splendid judgment. The wonderful development of the tooth business of this firm was due to his knowledge of that branch of the business. He learned the art of tooth carving from his father-in-law, Dr. Jefferies, of Philadelphia. Dr. Starr did other things as well as tooth carving. He is said to have taken out nearly one hundred patents on dental appliances of all kinds. The S. S. White cable engine is largely his invention. To many in the profession he was known personally, but thousands have and are reaping the benefits of his industry. It is well for us to stop and give thanks to the men, too often unseen, who do so much to lighten our labors, and are really benefactors of the highest type.

